# Impact of SARS-CoV2 infection on gut microbiota dysbiosis

**DOI:** 10.20517/mrr.2023.48

**Published:** 2023-12-06

**Authors:** Zhenming Xiao, Miaomiao Pan, Xinyao Li, Chao Zhao

**Affiliations:** ^1^Key Laboratory of Medical Molecular Virology, School of Basic Medical Sciences, Shanghai Medical College & National Clinical Research Center for Aging and Medicine, Huashan Hospital, Shanghai Medical College, Fudan University, Shanghai 200032, China.; ^2^Shanghai Frontiers Science Center of Pathogenic Microbes and Infection, Shanghai Frontiers Science Center, Shanghai 200032, China.

**Keywords:** COVID-19, gut microbiome, gut-lung axis, inflammation, immunity

## Abstract

The composition and function of the gut microbiota constantly influence health. Disruptions in this delicate balance, termed gut microbiota dysbiosis, have been implicated in various adverse health events. As the largest global epidemic since 1918, the severe acute respiratory syndrome coronavirus 2 (SARS-CoV-2) had devastating consequences. While the primary impact of Corona Virus Disease 2019 (COVID-19) has been on the respiratory system, a growing body of research has unveiled the significant involvement of the gastrointestinal tract as well. Emerging evidence underscores notable alterations in the gut microbiome of COVID-19 patients. In addition, the gut microbiome is also characterized by an abundance of opportunistic pathogens, which is related to disease manifestations of COVID-19 patients. The intricate bidirectional interaction between the respiratory mucosa and the gut microbiota, known as the gut-lung axis, emerges as a crucial player in the pathological immune response triggered by SARS-CoV-2. Here, we discuss microbiota-based gut characteristics of COVID-19 patients and the long-term consequences of gut microbiota dysregulation. These insights could potentially transform the development of long-term interventions for COVID-19, offering hope for improved outcomes and enhanced patient recovery.

## INTRODUCTION

The human gut microbiota plays a crucial role in maintaining our overall health, as it helps in digestion, absorption of nutrients, and the development of our immune system^[1]^. Any alteration in the gut microbiota can lead to various health problems, including inflammatory bowel disease, obesity, diabetes, and even mental disorders. This imbalance, known as gut microbiota dysbiosis, can be instigated by a variety of factors such as diet, age, medication, and infections^[2]^. Recent studies have shown that viral respiratory infections can cause gut microbiota dysbiosis by altering the microbial composition of the gut^[3]^. Conversely, changes in the gut microbiota can even affect the disease outcomes of distant organs, including the lung, which has been demonstrated by transfer experiments with dysbiosis of the gut microbiota^[4]^.

Corona Virus Disease 2019 (COVID-19) is an infectious disease first recognized in Wuhan, China, in December 2019^[5]^. The pandemic of COVID-19 was a global public health emergency caused by the widespread infection of severe acute respiratory syndrome coronavirus 2 (SARS-CoV-2) and the development of infectious respiratory diseases. While respiratory symptoms dominate the clinical syndromes of COVID-19 cases, a subset of patients grapple with concurrent gastrointestinal manifestations such as diarrhea and nausea. Individuals afflicted by COVID-19 manifest discernible alterations in gut microbiota. Upon hospitalization, their gut microbiota demonstrates an enrichment of opportunistic pathogens, coupled with a decline in beneficial commensals^[6]^.

This review explores the gut microbiota dysbiosis observed in patients who are infected with COVID-19 and plausible mechanisms culminating in this dysbiosis, unraveling the interplay of factors that orchestrate the microbial imbalance. Furthermore, it delves into the aftermath of COVID-19-induced gut microbiota dysbiosis, spotlighting its pivotal role in heightening the risk of systemic maladies. Overall, by shining a spotlight on the relationship between COVID-19 and gut microbiota, this review accentuates the imperative of comprehending its impact on human health and offers a stepping stone for potential interventions and safeguards.

## GUT MICROBIOTA ALTERATIONS IN PATIENTS INFECTED BY COVID-19

The gut microbiota is the densest bacterial community known to exist in the human body and plays an important metabolic and immune process in human health by metabolizing indigestible carbohydrates, producing vitamins, preventing pathogen infections, and modulating host immune responses^[7]^. Since the outbreak of COVID-19, extensive research has been conducted on its various symptoms and impacts. In addition to the main respiratory symptoms, more and more studies have shown that COVID-19 also affects the composition and function of human gut microbiota^[8,9]^.

Zuo *et al.*’s pioneering study revealed the disruption of gut microbiota in COVID-19 patients, highlighting stark differences in fecal microbial composition compared to healthy counterparts. Hospitalized COVID-19 patients exhibited an enrichment of bacteremia-associated microbial communities, accompanied by a decline in commensals beneficial to host immunity. The baseline abundances of *Coprobacillus, Clostridium ramosum*, and *Clostridium hathewayi* were positively correlated with the severity of COVID-19, while *Faecalibacterium prausnitzii* was negatively correlated with severity^[6]^. In another parallel study, Zuo *et al.* reported that fecal fungi of COVID-19 patients showed greater individual differences compared with healthy individuals, revealing higher heterogeneity. Compared with healthy individuals, patients infected by COVID-19 had increased proportions of opportunistic fungal pathogens, *Candida albicans*, *Candida auris*, *and Aspergillus flavus*^[10]^. Interestingly, COVID-19’s influence on gut microbiota is intertwined with changes in oral and respiratory microbiota. Some similarities were found in the changing patterns of the composition of respiratory, oral, and gut microbiota, but whether these patterns result from microbial transfer between microbiota across different locations remains unclear^[8,11]^.

Furthermore, there were differences in the gut microbiota of patients infected by COVID-19 compared with other pneumonia patients. These variations signify a unique microbial signature associated with COVID-19, even in cases with similar clinical presentations^[6]^. Although clinical symptoms were similar between H1N1-infected patients and COVID-19 patients, Gu *et al.* observed that a remarkably higher relative abundance of *Actinomycetota* and *Bacillota* is observed in patients infected by COVID-19 compared with 24 patients infected by H1N1 influenza^[12]^. Here, we summarize the gut microbiota dysbiosis in patients with COVID-19 in Table 1.

**Table 1:** The dysbiosis of gut microbiota in subjects infected with SARS-CoV-2

Wu *et al.* 2021^[8]^	Enriched	*Granulicatella*, *Rothia mucilaginosa*
He *et al.* 2021^[9]^	Enriched	*Bacteroides coprophilus*, *Bacteroides coprocola*, *Bacteroides graminisolvens*, *Bacteroides uniformis*
	Depleted	*Lachnospiraceae*, *Ruminococcus*, *Butyrivibrio*, *Dorea*, *Eubacterium*
Zuo *et al.* 2020^[6]^	Depleted	*Faecalibacterium prausnitzii*, *Lachnospiraceae bacterium 5_1_63FAA*, *Eubacterium rectale*, *Ruminococcus obeum*, *Dorea formicigenerans*
Zuo *et al.* 2020^[10]^	Enriched	*Candida albicans*, *Candida auris*, *Aspergillus flavus*, *Aspergillus niger*
Shen *et al.* 2022^[11]^	Enriched	*Enterococcus*, *Candida*
	Depleted	*Streptococcus*, *Actinomyces*, *Atopobium*, *Bacteroides*
Gu *et al.* 2020^[12]^	Enriched	*Actinomycetota*, *Bacillota*
Zhang *et al.* 2021^[13]^	Enriched	*Bifidobacterium adolescentis*, *Ruminococcus bromii*, *F prausnitzii*, *Bacteroides ovatus*, *Bacteroides dorei*, *Bacteroides thetaiotaomicron*
Lv *et al.* 2021^[14]^	Enriched	*Streptococcus*
	Depleted	*Peptostreptococcaceae*, *Penicillium steckii*, *Aspergillus rugulosus*

SARS-CoV-2: Severe acute respiratory syndrome coronavirus 2.

Moreover, emerging studies have shed light on the functional capacity of gut microbiota. The fecal metabolomic and proteomic profile in patients infected by COVID-19 is also illustrated in these studies. Studies have shown impaired short-chain fatty acid (SCFA) biosynthesis in fecal samples of patients infected by COVID-19^[6,13,14]^. Increased fecal sucrose and depleted fecal glucose levels were reported in patients infected by COVID-19 compared with other individuals without COVID-19^[15]^.

Post-COVID-19 syndrome (PCS), which includes prolonged clinical symptoms and comorbidities, is a common occurrence among COVID-19 rehabilitation patients^[16]^. Vestad *et al.* reported significant differences in gut microbiota between COVID-19 rehabilitation patients and healthy adults. COVID-19 rehabilitation patients had a trend of lower relative abundance of *Erysipelotrichaceae UCG-003* and higher relative abundance of *Veillonella* and *Flavonifractor* 3 months after recovery, and these taxa were strongly linked to persistent respiratory impairment^[17]^. Another follow-up study revealed that patients with a 1-year recovery period show gradual enrichment of butyric-producing bacteria (such as *Eubacterium*, *Faecalibacterium*, and *Bifidobacterium*), accompanied by an increase in α diversity of fecal microbiota and a lower abundance of lipopolysaccharide (LPS)-producing bacteria, such as *Intestinibacter* and *Prevotellaceae*^[18]^. Additionally, existing studies have reported that changes in the gut microbiota may persist for more than a year after recovery from COVID-19^[19]^.

## POTENTIAL MECHANISMS FOR GUT DYSBIOSIS IN COVID-19 PATIENTS

The importance of the gut-lung axis has gained increasing attention with the in-depth study of COVID-19. This axis orchestrates an intricate connection facilitated by the lymphatic system and blood circulation, ferrying immune cell mediators, cytokines, microbial fragments or products (peptidoglycan, endotoxins, proteins, SCFA, and hormones between these two vital systems)^[20]^. Although respiratory viral infection does not directly cause changes in the gut microbiota, lung inflammation can elicit systemic inflammatory signals and initiate local inflammatory responses in the gut, which appears to contribute to intestinal injury^[21]^. In addition, some evidence suggests that other viral infections in the lungs may affect the gut microbiota through the gut-lung axis by immune mediation. For example, influenza infection altered the intestinal microbiota composition, which was mediated by IFN-γ produced by lung-derived CCR9^+^CD4^+^ T cells recruited into the small intestine^[20]^. Respiratory syncytial virus (RSV) infection reduced host food consumption and altered gut microbiota by stimulating the proliferation of CD8^+^ T cells secreting IL-6 and TNF-α^[22]^. Invasion of SARS-CoV-2 into the lungs could damage tissue and cells and activate strong pro-inflammatory pathways characterized by upregulation of NF-κB and TNF pathways to induce the intense cytokine storm^[23]^, while activated systemic and intestinal inflammation may be responsible for changes in the gut microbiota^[24]^. Briefly, invasion by SARS-CoV-2 into the lungs incites tissue damage to some extent and robust pro-inflammatory responses, potentially triggering cascading changes in gut microbiota through systemic and intestinal inflammation.

Significantly, mounting evidence points towards direct infection of the gastrointestinal tract by SARS-CoV-2. A single-cell RNA sequencing showed that ACE2 and transmembrane protease serine 2 (TMPRSS2) were highly expressed in gastrointestinal epithelial cells^[25,26]^. They were crucial for the entry of SARS-CoV-2 into the host cells, and their distribution determined the virus development^[27,28]^. More conclusive evidence of the correlation between COVID-19 infections and pathological changes in gastrointestinal systems is the large proportion of positive fecal samples for viral RNA in patients infected by COVID-19^[29]^. Furthermore, SARS-CoV-2 is often found in fecal samples during the post-symptom stage, even when throat swabs are negative^[30]^. Multiple histopathological examinations have also confirmed that SARS-CoV-2 presents gastrointestinal tropism. Coronavirus-like particles have been found in gastrointestinal tissues, and gastrointestinal mucosa has shown varying degrees of degeneration, necrosis, and shedding^[31]^. In studies of organoids and animal models (rhesus monkeys), the virus could infect the gastrointestinal tract^[32,33]^. Once the virus reaches the epithelial cells of the intestine, the cells will be bound to it via ACE-2, which can cause the release of chemokines and cytokines. Subsequently, the development of an inflammatory cascade in the intestine is characterized by the infiltration of macrophages, neutrophils, and T cells^[34]^. Another theory shows that SARS-CoV-2 disease downregulates ACE2, resulting in decreased activation of the mammalian target of rapamycin (mTOR) and increased autophagy, resulting in intestinal dysbiosis, diarrhea, and other sympotoms^[35]^. Recent studies indicate that specific microbial species such as *Bacteroides dorei*, *Bacteroides thetaiotaomicron*, *Bacteroides massiliensis*, and *Bacteroides ovatus* exhibit correlations with ACE2 expression and viral load, linking them to COVID-19 pathogenesis^[36]^.

The critical illness phase of COVID-19 is marked by a confluence of factors, including antibiotic usage, mechanical ventilation, diet shifts, emotional stress, and inflammatory responses, which can all perturb the delicate microbiome balance. In sicker patients, they often require prolonged mechanical ventilation and treatment with invasive catheters. These treatments increase susceptibility to secondary infections with multidrug-resistant pathogens such as *Acinetobacter baumannii*, *Escherichia coli*, and *Pseudomonas aeruginosa*^[37]^. In a study among the 99 cases of 2019 SARS-CoV-2 in Wuhan, the co-infecting pathogens include *Acinetobacter baumannii*, *Klebsiella pneumoniae*, and many others^[38]^. The relative abundance of these species in the intestines of patients infected by COVID-19 is higher than that of healthy individuals. The infection of these fungi has also increased the number of patients admitted to Intensive Care Unit (ICU) facilities and antibiotic treatments. Research suggests that about half of the deaths of hospitalized patients infected by COVID-19 are attributable to multidrug-resistant bacteria and fungus infections^[39]^. It is worth noting that microbial co-infection is a serious factor in COVID-19, exacerbating the processes of the occurrence, development and prognosis of COVID-19, and the difficulties of clinical diagnosis and treatment^[40]^.

Therefore, it is essential to consider the complex interplay of factors that may impact the gut microbiota in COVID-19 patients, including the direct impact of the virus, systemic inflammation, and treatment-related factors.

## CONSEQUENCES OF GUT MICROBIOTA DYSBIOSIS IN COVID-19 PATIENTS

A healthy gut microbiota is significantly necessary to maintain the human body’s immune homeostasis. Dysbiosis of the microbiota caused by COVID-19 infection can disrupt host immunity in the following ways. Butyric-producing bacteria can downregulate genes linked to SARS-CoV-2 infection^[41]^. SCFAs, including butyric acid, can promote CD8^+^ T cell function to facilitate the clearance and depletion of the influenza virus^[42]^. In addition, the differentiation of Treg cells and IL-10-producing T cells is stimulated by SCFAs binding to GPR109A, a butyrate receptor , which imparts an anti-inflammatory effect^[43]^. However, a paradox arises as SCFA-producing microbiota exhibit an inverse correlation with SARS-CoV-2 fecal abundance^[44]^. A previous study suggested that the *Bacteroidetes* phylum, which is reduced in severe and critically ill patients infected by COVID-19, could activate colonic dendritic cells through the TLR4-TRIF pathway^[45]^. *Lactobacillus*, which was proved to be remarkably decreased in COVID-19 patients^[46]^, can produce the aryl hydrocarbon receptor (AhR) ligand-indole-3-aldehyde that promotes AhR-dependent IL22 transcription and improves resistance against mucosal inflammation^[47]^. COVID-19 enrichment-induced opportunistic pathogen Veillonella is associated with Th17 cell recruitment, neutrophil enrichment, and IL7 inflammatory phenotype activation^[48]^.

The academic community widely acknowledges the potential for gut microbiota alterations to influence susceptibility to SARS-CoV-2^[49]^. Transferring gut bacteria from patients with long COVID to healthy mice resulted in lost cognitive functioning and impaired lung defenses in the mice^[50]^. Furthermore, the altered intestinal barrier of viral infection results in changes in the gut microbiota and its metabolites, and translocation of intestinal bacteria to the circulation or other sites, leading to increased systemic or local inflammation and subsequent impairment of multi-organ function^[51]^. The gut microbiota, serving as a pivotal connection among organs, is critical for maintaining the host system's equilibrium. Dysbiosis, observed in COVID-19 patients, escalates the risk of systemic disease manifestation.

In conditions of viral enteric infection, microorganisms and damaged tissue in the body can degrade and become a new source of nutrition, leading to a temporary oversupply of nutrition. This nutritional oversupply induces inflammation during acute infection^[52]^ and potentially leads to chronic diseases like malignancies^[53]^. These insights lay the foundation for further research into the relationship between gut microbiota alteration due to COVID-19 and the emphasized risk of developing or progressing colorectal cancer^[54]^. SCFAs regulate inflammation by macrophages in the intestine and promote the Warburg effect, which metabolically constrains the neoplastic cells; however, fecal samples from patients with high SARS-CoV-2 infectivity had lower abundances of SCFA-producing bacteria^[55]^. A reduction in SCFA-producing bacteria can also disrupt gut epithelial barrier integrity via IL-22 signaling and promote inflammatory signaling through IL-18^[56,57]^.

**Figure 1:**
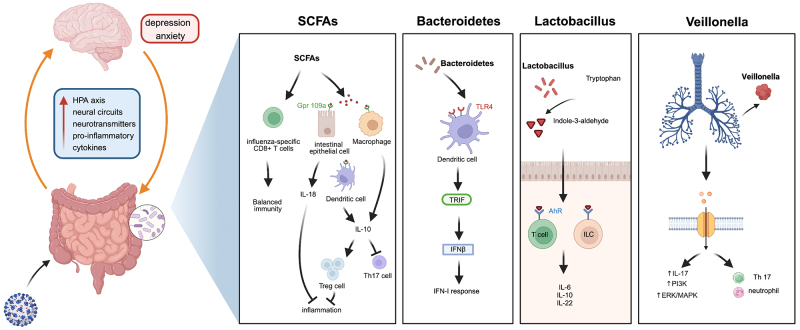
Dysbiosis of the gut microbiota caused by SARS-CoV-2 infection interferes with host immunity and mental health. AhR: Aryl hydrocarbon receptor; SARS-CoV-2: severe acute respiratory syndrome coronavirus 2; SCFA: short-chain fatty acid.

In addition, the relationship between gut microbiota and mental illness is a hot topic of current research^[58,59]^. The gut-brain axis, facilitating communication between the gut and the brain, emerges as a pivotal consideration in gut microbiota research. Numerous studies have shown that dysbiosis has been linked to mental illnesses such as depression and anxiety, disrupting the gut-brain axis^[60]^. Ghannoum *et al.* reported that altered intestinal flora in the multiple dimensions of the gut-brain axis had a negative impact on the body, including excessive activation of the hypothalamic-pituitary-adrenal (HPA) axis, neural circuits, and the level of neurotransmitters^[61]^. Eventually, depression and anxiety result from these changes. Nakov *et al.* reported an increased prevalence of gastrointestinal symptoms during the COVID-19 lockdown^[62]^. They found that these symptoms were linked to dysfunction of the gut-brain axis, thereby causing alterations in the neuroimmune and endocrine systems and promoting the negative development of depression and anxiety. A long-term prospective study confirmed that neuropsychiatric symptoms were associated with enteric pathogens, including Clostridium innocuum and Actinomyces naeslundii, in the long-term complications of COVID-19^[63]^. Collectively, changes in the gut microbiome and microbes that produce specific metabolites, such as butyrate, may contribute through the gut-brain axis to neurological and psychiatric symptoms as well as GI symptoms in COVID-19 patients^[59]^. The possible mechanism of dysbiosis caused by SARS-CoV-2 infection on host immunity and mental health is illuminated in Figure 1.

The intricate interplay between SARS-CoV-2, the gut microbiota, and its implications for immunity, disease susceptibility, and mental health unfolds a multifaceted narrative. Exploring these connections offers a promising avenue for advancing our comprehension of COVID-19’s profound effects.

## CONCLUDING REMARKS AND PROSPECTS

COVID-19 has demonstrated a notable impact on the intricate composition and functioning of the gut microbiota. Studies have shown differences in fecal microbial composition between healthy individuals and COVID-19 patients, with an enrichment of bacteremia-associated microbial communities and a reduction in beneficial commensals. The severity of COVID-19 has been found to correlate with specific bacterial abundances, thereby underscoring the intricate relationship between microbial composition and disease severity. Additionally, COVID-19 patients’ fecal fungi have shown remarkable heterogeneity, further accentuating the complex nature of gut microbiota response. The ripple effects of COVID-19 transcend the gut microbiota alone, influencing the composition of both respiratory and oral microbiota. Additionally, individuals afflicted by COVID-19 exhibit a gut microbiota profile distinct from that of patients with other forms of pneumonia, highlighting the unique microbial interactions associated with this viral infection. The dysbiosis of the gut microbiota in COVID-19 patients is thought to be influenced by various factors, including the gut-lung axis, direct infection of the gastrointestinal tract by SARS-CoV-2, and critical illness interventions such as antibiotic use and mechanical ventilation. This intricate interplay contributes to an altered immune response and heightened susceptibility to secondary infections. It has been observed that alterations in carbohydrate, lipid, and amino acid metabolism of the gut microbiota are associated with COVID-19. Post-COVID-19 syndrome, also known as long COVID, can also impact the gut microbiota, with persistent changes observed even after recovery. The repercussions of gut microbiota dysbiosis in the context of COVID-19 are profound. Host immunity is compromised, inflammation escalates, and the risk of systemic diseases amplifies. An ominous link emerges between dysbiosis and the potential for colorectal cancer development or progression. Beyond the physiological realm, the perturbations in the gut-brain axis provoke mood disorders such as depression and anxiety.

In outlook, further research is needed to understand the mechanisms underlying the gut microbiota shifts in COVID-19 patients and their implications for disease severity and long-term outcomes. This knowledge holds the potential to not only illuminate the relationship between microbial composition and disease severity but also to offer insights for innovative therapeutic strategies. Probing the composition of the microbiota and its metabolites in the context of an emerging infectious disease may unveil new biomarkers and new therapeutic targets. The growth of this field is evident through milestones achieved during the pandemic, ranging from gut microbial data mining to clinical applications. With the development of multi-omics of microorganisms and the application of novel microbial therapeutics, new discoveries in the microbiota are expected to be a powerful aid in the fight against emerging infectious diseases.

## DECLARATIONS

### Copyright

© The Author(s) 2023.
